# Data from renewable energy assessments for resort islands in the South China Sea

**DOI:** 10.1016/j.dib.2015.11.043

**Published:** 2015-11-25

**Authors:** M. Reyasudin Basir Khan, Razali Jidin, Jagadeesh Pasupuleti

**Affiliations:** College of Engineering, Universiti Tenaga Nasional, Jalan IKRAM – UNITEN, 43000 Kajang, Selangor, Malaysia

**Keywords:** South China Sea, Solar radiation,wind speed, rainfall, microhydropower, PV system, Wind energy generation system

## Abstract

Renewable energy assessments for resort islands in the South China Sea were conducted that involves the collection and analysis of meteorological and topographic data. The meteorological data was used to assess the PV, wind and hydropower system potentials on the islands. Furthermore, the reconnaissance study for hydro-potentials were conducted through topographic maps in order to determine the potential sites suitable for development of run-of-river hydropower generation. The stream data was collected for 14 islands in the South China Sea with a total of 51 investigated sites. The data from this study are related to the research article *“Optimal combination of solar, wind, micro-hydro and diesel systems based on actual seasonal load profiles for a resort island in the South China Sea”* published in Energy (Khan et al., 2015) [1].

**Specifications Table**

**Table:** 

Subject area	*Physics*
More specific subject area	*Meteorology; Energy*
Type of data	*Table, figure*
How data was acquired	*Malaysian Meteorological Department.*
*Department of Survey and Mapping Malaysia.*
Data format	*Filtered*
Experimental features	*Meteorological data extrapolation based on* linear extrapolation technique *using Matlab software.*
*Topographic map data extraction based on hydropower guidelines.*
Data source location	*South China Sea Islands, East Coast of Peninsular Malaysia*
Data accessibility	*Data is provided in supplementary materials directly with this article*

**Value of the data**

• The data describes the meteorological and topographical conditions of the resort islands in the South China Sea
• This data contains key information for renewable energy assessments for resort islands in the South China Sea.
• This data can be used for other research fields that involve the usage of solar radiation, wind speed, rainfall and evaporation data.
• The topographic map data is valuable for determining the potential run-of-river hydropower sites in many resort islands in the South China Sea.

## 1 Data

The data consists of meteorological and topographical data for resorts islands in the South China Sea.

**Fig. 1:**
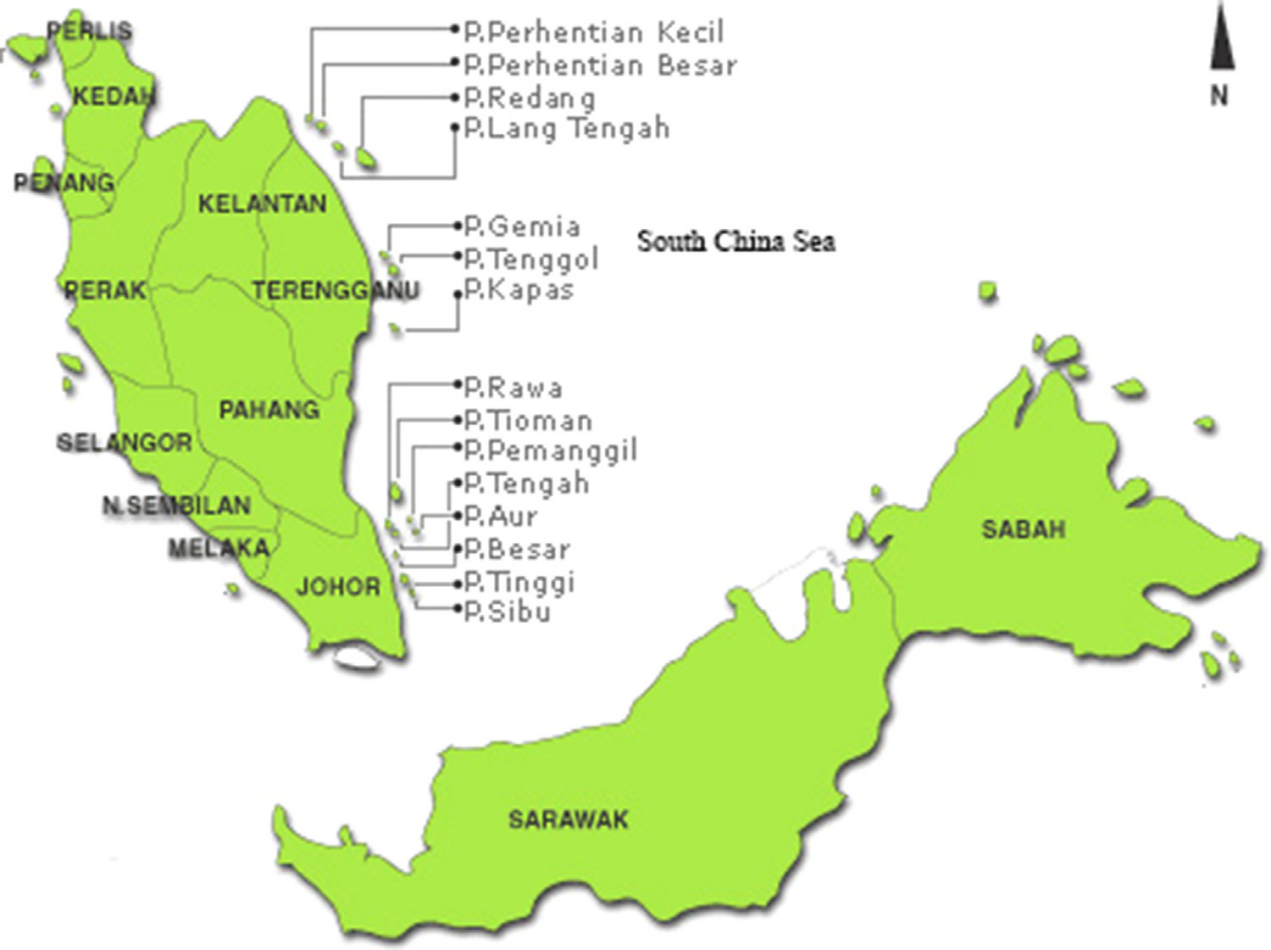
Selected islands in the South China Sea.

The selected resort islands location is shown in Fig. 1. The meteorological data consists of daily mean wind speed, daily rainfall, daily solar radiation and daily evaporation for a period of three years (2005–2007). Meanwhile the topographic map data consists of the site׳s stream flow parameters such as location, stream name, available head, catchment area and river gradient. Several other data such as cultural features, miscellaneous constructions and vegetation type on the sites were also included in the data [1].

## 2 Experimental design, materials and methods

The meteorological data for the resort islands was obtained from the Malaysia Meteorological Department (MMS) and also the NASA Prediction of Worldwide Energy Resource (NASA POWER) database. If there is no meteorological station on the island, the meteorological data was collected from the nearest available meteorological station. The nearest station information׳s also included in this article Supplementary material. The meteorological stations selected based on its distance with the island which has been estimated using Google Earth. There were also many missing data in the meteorological data; hence, the missing data has been filled based on a linear extrapolation technique using Matlab software.

The topographic map obtained from Department of Survey and Mapping Malaysia (DSMM) was used for the study. The map is classified and must be used within the DSMM map library. The characteristic of the map used for this research is a Malaysian topography map (Series L 7030) with 1:50,000 scales and a contour interval of 20 m. The physiographic characteristics were extracted from the map for prediction of run-of-river hydropower potential sites. The important information extracted and analyzed from the map were: the name of streams and catchment areas, latitude and longitude of the location, lowest and highest elevation, the terrain and river profile, possible intake, diversion to fore bay, power house elevation, and the estimation of available head and catchment areas.

Three key factors were considered for selecting catchment areas from a topographic map that were suitable for harnessing hydropower. The key factors were energy demand, accessibility and river profile. The catchment areas topographies were studied for determining the appropriate elevation for head and stream diversion. The river profile, which is the river׳s tributaries and gradient, was considered for finding the availability of water resources and river flow. Based on the hydropower manual and guides [2], [3], [4], [5], the catchment areas, streams, available heads and river profile that were suitable for hydropower development were identified from the topographic map.
