# Supplementary material for: Data from renewable energy assessments for resort islands in the South China Sea
Source: Data Brief. 2015 Nov 25;6:117–20. doi: 10.1016/j.dib.2015.11.043 (PMC4685173; doi:10.1016/j.dib.2015.11.043)
Supplement: Supplementary file 1 — Supplementary material [file mmc1.zip › Islands and meteorological station data.docx]

Islands Description

| Island | Location | Coordinate | Area (km^2^) |
| --- | --- | --- | --- |
| Pulau Perhentian Kecil | Terengganu | 5°54'25"N 102°43'23"E | 5.7 |
| Pulau Perhentian Besar | Terengganu | 5°54'5"N 102°45'22"E | 9.2 |
| Pulau Redang | Terengganu | 5°46'28"N 103° 0'34"E | 23.4 |
| Pulau Lang Tengah | Terengganu | 5°47'45"N 102°53'45"E | 1.2 |
| Pulau Gemia | Terengganu | 5°14'N 103°15'47"E | 0.05 |
| Pulau Tenggol | Terengganu | 4°48'15"N 103°40'52"E | 2.6 |
| Pulau kapas | Terengganu | 5°13'13"N 103°15'55"E | 1.4 |
| Pulau Rawa | Johor | 2°31'15"N 103°58'33"E | 0.2 |
| Pulau Tioman | Pahang | 2°47'47"N 104°10'24"E | 138 |
| Pulau Pemanggil | Johor | 2°34'51"N 104°19'39"E | 8.6 |
| Pulau Aur | Johor | 2°26'60"N 104°31'9"E | 14.2 |
| Pulau Besar | Johor | 2°26'22"N 103°58'52"E | 4.6 |
| Pulau Tinggi | Johor | 2°17'56"N 104°7'2"E | 15 |
| Pulau Sibu | Johor | 2°13'3"N 104° 4'16"E | 4 |

| Stations | Islands | Distance (Km) |
| --- | --- | --- |
| 48674 Mersing Meterological Station (Johor)  Coordinate: 2°27'N 103°50'E  2.45N 103.83E  43.6 above MSL | Pulau Rawa | 17.7 |
|  | Pulau Tioman | 53.8 |
|  | Pulau Pemanggil | 56.8 |
|  | Pulau Aur | 76.3 |
|  | Pulau Besar | 16.5 |
|  | Pulau Tinggi | 35.7 |
|  | Pulau Sibu | 36.9 |
| Stations | Islands | Distance (Km) |
| 48618 Kuala Terengganu Meteorological Station (Terengganu)  Coordinate: 5°23'N 103°06'E  5.383N 103.1E  5.0m above MSL | Pulau Perhentian Kecil | 71.4 |
|  | Pulau Perhentian Besar | 68.8 |
|  | Pulau Redang | 44.4 |
|  | Pulau Lang Tengah | 50.9 |
|  | Pulau Gemia | 24.5 |
|  | Pulau Tenggol | 90.9 |
|  | Pulau kapas | 25.7 |
| Stations | Islands | Distance (Km) |
| 48615 Kota Bharu Meteorological Station (Kelantan)  Coordinate: 6°10'N 102°17'E  6.167N 102.283E  4.6m above MSL | Pulau Perhentian Kecil | 56.5 |
|  | Pulau Perhentian Besar | 60 |
|  | Pulau Redang | 91.3 |
|  | Pulau Lang Tengah | 79.2 |
|  | Pulau Gemia | 149.8 |
|  | Pulau Tenggol | 216.1 |
|  | Pulau kapas | 151 |

| Island | Location | Coordinate | Nearest meteorological Station | Distance  (Km) |
| --- | --- | --- | --- | --- |
| Pulau Perhentian Kecil | Terengganu | 5°54'25"N 102°43'23"E | Kota Bharu Meteorological Station  (Kelantan) | 56.5 |
| Pulau Perhentian Besar | Terengganu | 5°54'5"N 102°45'22"E | Kota Bharu Meteorological Station  (Kelantan) | 60.0 |
| Pulau Redang | Terengganu | 5°46'28"N 103° 0'34"E | Kuala Terengganu Meteorological Station (Terengganu) | 44.4 |
| Pulau Lang Tengah | Terengganu | 5°47'45"N 102°53'45"E | Kuala Terengganu Meteorological Station (Terengganu) | 50.9 |
| Pulau Gemia | Terengganu | 5°14'N 103°15'47"E | Kuala Terengganu Meteorological Station (Terengganu) | 24.5 |
| Pulau Tenggol | Terengganu | 4°48'15"N 103°40'52"E | Kuala Terengganu Meteorological Station (Terengganu) | 90.9 |
| Pulau kapas | Terengganu | 5°13'13"N 103°15'55"E | Kuala Terengganu Meteorological Station (Terengganu) | 25.7 |
| Pulau Rawa | Johor | 2°31'15"N 103°58'33"E | Mersing Meterological Station (Johor) | 17.7 |
| Pulau Tioman | Pahang | 2°47'47"N 104°10'24"E | Mersing Meterological Station (Johor) | 53.8 |
| Pulau Pemanggil | Johor | 2°34'51"N 104°19'39"E | Mersing Meterological Station (Johor) | 56.8 |
| Pulau Aur | Johor | 2°26'60"N 104°31'9"E | Mersing Meterological Station (Johor) | 76.3 |
| Pulau Besar | Johor | 2°26'22"N 103°58'52"E | Mersing Meterological Station (Johor) | 16.5 |
| Pulau Tinggi | Johor | 2°17'56"N 104°7'2"E | Mersing Meterological Station (Johor) | 35.7 |
| Pulau Sibu | Johor | 2°13'3"N 104° 4'16"E | Mersing Meterological Station (Johor) | 36.9 |

| Island | Location | Central Coordinate | Coordinate for data collection | Coordinate in Decimal Degree | Height above sea level (m) |
| --- | --- | --- | --- | --- | --- |
| Pulau Perhentian Kecil | Terengganu | 5°54'25"N 102°43'23"E | 5°53'51"N 102°43'46"E | 5.898N 102.729E | 8 |
| Pulau Perhentian Besar | Terengganu | 5°54'5"N 102°45'22"E | 5°53'38"N 102°44'50"E | 5.894N 102.747E | 10 |
| Pulau Redang | Terengganu | 5°46'28"N 103° 0'34"E | 5°46'16"N 103° 1'58"E | 5.771N 103.033E | 9 |
| Pulau Lang Tengah | Terengganu | 5°47'45"N 102°53'45"E | 5°47'20"N 102°53'57"E | 5.789N 102.899E | 12 |
| Pulau Gemia | Terengganu | 5°14'N 103°15'47"E | 5°13'56"N 103°15'43"E | 5.232N 103.262E | 10 |
| Pulau Tenggol | Terengganu | 4°48'15"N 103°40'52"E | 4°48'31"N 103°40'43"E | 4.809N 103.679E | 12 |
| Pulau kapas | Terengganu | 5°13'13"N 103°15'55"E | 5°13'12"N 103°15'40"E | 5.220N 103.261E | 8 |
| Pulau Rawa | Johor | 2°31'15"N 103°58'33"E | 2°31'4"N 103°58'34"E | 2.512N 103.976E | 13 |
| Pulau Tioman | Pahang | 2°47'47"N 104°10'24"E | 2°47'10"N 104° 7'13"E | 2.786N 104.120E | 5 |
| Pulau Pemanggil | Johor | 2°34'51"N 104°19'39"E | 2°34'53"N 104°18'59"E | 2.581N 104.316E | 8 |
| Pulau Aur | Johor | 2°26'60"N 104°31'9"E | 2°27'9"N 104°30'10"E | 2.453N 104.503E | 14 |
| Pulau Besar | Johor | 2°26'22"N 103°58'52"E | 2°26'N 103°58'42"E | 2.433N 103.978E | 9 |
| Pulau Tinggi | Johor | 2°17'56"N 104°7'2"E | 2°17'37"N 104° 6'10"E | 2.294N 104.103E | 12 |
| Pulau Sibu | Johor | 2°13'3"N 104° 4'16"E | 2°12'50"N 104° 4'34"E | 2.214N 104.076E | 7 |

Coordinate selected for NASA data collection
