# Supplementary material for: Data from renewable energy assessments for resort islands in the South China Sea
Source: Data Brief. 2015 Nov 25;6:117–20. doi: 10.1016/j.dib.2015.11.043 (PMC4685173; doi:10.1016/j.dib.2015.11.043)
Supplement: Supplementary file 1 — Supplementary material [file mmc1.zip › topographic map data .docx]

1. **Pulau Tioman**

Mountains and Hills.

- Mount Kajang (1038m) –Trigonometry station
- Seperuk Hill (958m)- Trigonometry station
- Parang Panjang Hill (488m)
- Kerayung Besar Hill (409m)
- Kerayung kecil Hill (390m)

1. Kg Tekek ( 2° 49’ 00” N, 104° 10’ 00”E)

- Police station, postal Agency, Hospital, School, Airport, 2 water tank, 3 wooden bridge, 1 mosque, 1 wireless mast
- Area = ( 2.9 × 1.4) + ( 0.9 × 2) +( 1 × 1) +( 0.9 × 1.4)

= 4.06 + 1.8 + 1 + 1.26

= 8.12cm^2^

= 2.03km^2^

- Vegetation:

1. Secondary jungle, Belukar, Riung and Jerami
2. Rubber
3. Coconut
4. Sundry tree cultivation
5. S. Tekek

- Highest elevation: 440m
- Possible intake : 260m ( 2° 49’ 11” N, 104° 10’ 34”E)
- Catchment area = 1.1 ×1.1

= 1.21cm^2^

= 0.303km^2^

- Single line river
- Footpath at height below 20m
- Flows from Bukit Parang Panjang (488m)
- Tailrace= 80m
- Available head = 260-80

= 180m

- River gradient = $\frac{260}{1550}$

= 0.168

1. S. Air Besar

- Highest elevation: 700m
- Possible intake : 280m ( 2° 48’ 57” N, 104° 10’ 28”E)
- Catchment area = (0.7 ×1.2) + (0.45 ×1.1) +(1.5 ×2.5) + (0.7 ×1.4) + (1.2 ×1.3)

= 0.84 + 0.495 + 3.75 + 0.98 + 1.56

= 7.625cm^2^

= 1.906km^2^

- Single line river
- Tailrace= 40m
- Available head = 280-40

= 240m

- River gradient = $\frac{280}{1600}$

= 0.175

1. S. Batang Sabut

- Highest elevation: 440m
- Possible intake : 140m ( 2° 48’ 39” N, 104° 9’ 45”E)
- Catchment area = (1 ×1.1) + (0.5 ×0.8)

= 1.1 + 0.4

= 1.5cm^2^

= 0.375km^2^

- Single line river
- Tailrace= 40m
- Available head = 140-40

= 100m

- River gradient = $\frac{140}{1.9cm \times\frac{1km}{2cm}\times\frac{1000 m}{1 km}}$ =$\frac{140}{950}$ = 0.147

1. S. Tg. Said

- Highest elevation: 320m
- Possible intake : 240m ( 2° 48’ 28” N, 104° 9’ 26”E)
- Catchment area = (0.7 ×0.55)

= 0.385cm^2^

= 0.0963km^2^

- Single line river
- Tailrace= 40m
- Available head = 240-40

= 200m

- River gradient = $\frac{240}{2cm \times\frac{1km}{2cm}\times\frac{1000 m}{1 km}}$ =$\frac{240}{1000}$ = 0.24

1. Kg Lalang ( 2° 48’ 25” N, 104° 08’ 55”E)

- Tioman Island Resort. 1 water tank
- Area = ( 1.5 × 1.9) + ( 0.6 × 0.7)

= 2.85 + 0.42

= 3.27cm^2^

= 0.817km^2^

- Vegetation:

1. Secondary jungle, Belukar, Riung and Jerami
2. Sundry tree cultivation
3. S. lalang

- Highest elevation: 900m
- Possible intake : 180m ( 2° 48’ 8” N, 104° 09’ 2”E)
- Catchment area = (1 ×1) + (1.6 ×1.3) +(1.2 ×1.4)

= 1 + 2.08 + 1.68

= 4.76cm^2^

= 1.19km^2^

- Single line river
- Tailrace= 60m
- Available head = 180-60

= 120m

- River gradient = $\frac{180}{2.4cm \times\frac{1km}{2cm}\times\frac{1000 m}{1 km}}$ =$\frac{180}{1200}$ = 0.15

1. Kg Bunut ( 2° 47’ 53” N, 104° 07’ 55”E)

- Wireless mast, low energy demand
- Vegetation:

1. Secondary jungle, Belukar, Riung and Jerami

S. Bunut

- No hydro potential , no energy demand

1. S. Setup

- Highest elevation: 520m
- Possible intake : 200m ( 2° 47’ 49” N, 104° 08’ 23”E)
- Catchment area = (1.3 ×1.9)

= 2.47cm^2^

= 0.618km^2^

- Single line river
- Tailrace= 20m
- Available head = 200-20

= 180m

- River gradient = $\frac{200-10}{1.4cm \times\frac{1km}{2cm}\times\frac{1000 m}{1 km}}$ =$\frac{190}{700}$ = 0.271

1. Kg Paya ( 2° 47’ 08” N, 104° 07’ 32”E)

- Area = ( 1.5 × 0.6) + ( 0.9 × 0.7)

= 0.9 + 0.54

= 1.44cm^2^

= 0.36km^2^

- Vegetation:

1. Secondary jungle, Belukar, Riung and Jerami
2. S. Paya

- Highest elevation: 880m
- Possible intake : 240m ( 2° 46’ 58” N, 104° 08’ 23”E)
- Catchment area = (1.1 ×1) + (1.9 ×2.4) +(1.1 ×3.6)

= 1 + 4.56 + 3.96

= 9.62cm^2^

= 2.4km^2^

- Single line river
- Tailrace= 20m
- Available head = 240-20

= 220m

- River gradient = $\frac{240}{4.8cm \times\frac{1km}{2cm}\times\frac{1000 m}{1 km}}$ =$\frac{240}{2400}$ = 0.1

1. Kg Genting ( 2° 45’ 47” N, 104° 07’ 37”E)

- Community hall. Clinic,mosque
- Area = ( 0.7 × 1) + ( 0.4 × 0.3) + ( 1.4 × 0.4) + ( 1.8 × 0.5) + ( 0.5 × 0.8) + ( 0.8 × 0.3)

= 0.7 + 0.12 + 0.56 + 0.9 + 0.4 + 0.24

= 2.92cm^2^

= 0.73km^2^

- Vegetation:

1. Secondary jungle, Belukar, Riung and Jerami
2. Sundry tree cultivation

- S. Air Genting
- No hydropower potential, no tributaries, no confluence

1. S. Air Jerangau

- Low tributaries, only one tributary and one confluence
- Highest elevation: 320m
- Possible intake : 200m ( 2° 46’ 12” N, 104° 07’ 38”E)
- Catchment area = (0.7 ×0.7)

= 0.49 cm^2^

= 0.1225 km^2^

- Single line river
- Tailrace= 20m
- Available head = 200-20

= 180m

- River gradient = $\frac{200-4}{1.2cm \times\frac{1km}{2cm}\times\frac{1000 m}{1 km}}$ =$\frac{196}{600}$ = 0.33

1. S. Air Raja

- Highest elevation: 900m
- Possible intake : 160m ( 2° 45’ 56” N, 104° 07’ 54”E)
- Catchment area = (0.6 ×0.6) + (1.9 ×2.4) +(1.6 ×1.1)

= 0.36 + 4.56 + 1.76

= 6.68cm^2^

= 1.67km^2^

- Single line river
- Tailrace= 40m
- Available head = 160-40

= 120m

- River gradient = $\frac{160}{2cm \times\frac{1km}{2cm}\times\frac{1000 m}{1 km}}$ =$\frac{160}{1000}$ = 0.16

1. S. Air Gong

- Highest elevation: 820m
- Possible intake : 180m ( 2° 45’ 39” N, 104° 08’ 03”E)
- Catchment area = (0.7 ×0.4) + (1.1 ×0.7) +(0.3 ×1) ) +(1.2 ×1.3)

= 0.28 + 0.77 + 0.3+ 1.56

= 2.91cm^2^

= 0.728km^2^

- Single line river
- Tailrace= 40m
- Available head = 180-40

= 140m

- River gradient = $\frac{180}{1.7cm \times\frac{1km}{2cm}\times\frac{1000 m}{1 km}}$ =$\frac{180}{850}$ = 0.21
- S. Air Taling
- No hydro potential, no tributaries, confluence, small river
- S. Mangkai
- No hydro potential, available head < 20m

*S Air Gong, S Air Taling and S Mangkai flows into the same river stem*

- S. Air Carut
- No hydro potential

1. Tg Permata ( 2° 44’ 43” N, 104° 07’ 29”E)

- Area = ( 0.5 × 0.5)

= 0.25cm^2^

= 0.0625km^2^

- Vegetation:

1. Secondary jungle, Belukar, Riung and Jerami

- S. Ronot
- No hydro potential, small river, available head < 20m

1. Tg K. Nipah Tg Kasut- Tg K Nipah ( 2° 44’ 28” N, 104° 08’ 05”E)

- Area = ( 0.6 × 0.5)

= 0.3cm^2^

= 0.075km^2^

- Vegetation:

1. Coconut
2. S. Kasut

- Highest elevation: 280m
- Possible intake : 180m ( 2° 44’ 43” N, 104° 08’ 03”E)
- Catchment area = (0.9 ×1.2)

= 1.08cm^2^

= 0.27km^2^

- Single line river
- Tailrace= 40m
- Available head = 180-40

= 140m

- River gradient = $\frac{180}{1.1cm \times\frac{1km}{2cm}\times\frac{1000 m}{1 km}}$ =$\frac{180}{550}$ = 0.33

1. S. Nipah

- Highest elevation: 940m
- Possible intake : 210m ( 2° 44’ 54” N, 104° 08’ 36”E)
- Catchment area = (0.7 ×1.2) + (0.4 ×1.2) +(0.7 ×2.1) + (0.5 ×2.4) +(0.6 ×0.4) + (1.5 ×0.5) +(2.8 ×2.7)

= 0.84 + 0.48 + 1.47 +1.2 + 0.24+ 0.75 + 7.56

= 12.54cm^2^

= 3.14km^2^

- Single line river
- Tailrace= 40m
- Available head = 210-40

= 170m

- River gradient = $\frac{210-10}{2.3cm \times\frac{1km}{2cm}\times\frac{1000 m}{1 km}}$ =$\frac{200}{1150}$ = 0.17

1. S. Seriah

- Highest elevation: 800m
- Possible intake : 120m ( 2° 44’ 36” N, 104° 08’ 42”E)
- Catchment area = (1.8 ×3.5) + (0.2 ×0.8) +(1.1 ×0.2) + (1.5 ×4) +(1 ×1.7) + (0.7 ×4) +(0.7 ×2.1)

= 6.3 + 0.16 + 0.22 +6 + 1.7+ 2.8 + 1.47

= 18.65cm^2^

= 4.66km^2^

- Single line river
- Tailrace= 20m
- Available head = 120-20

= 100m

- River gradient = $\frac{120-10}{2.2cm \times\frac{1km}{2cm}\times\frac{1000 m}{1 km}}$ =$\frac{110}{1100}$ = 0.1
- S Seriah and S Nipah flows into the same bay. Both of the river has the potential for mini hydro development in the future since it has very large catchment area, potential for higher head and several option for hydropower scheme.

1. Kg Pasir Sarang Burung ( 2° 43’ 20” N, 104° 11’ 10”E)

- Area = NA
- Vegetation:

1. Secondary jungle, Belukar, Riung and Jerami
2. S. Tedau

- Highest elevation: 580m
- Possible intake : 200m ( 2° 43’ 36” N, 104° 09’ 24”E)
- Catchment area = (2 ×2.1)

= 4.2cm^2^

= 1.05km^2^

- Single line river
- Tailrace= 20m
- Available head = 200-20

= 180m

- River gradient = $\frac{200}{1.6cm \times\frac{1km}{2cm}\times\frac{1000 m}{1 km}}$ =$\frac{200}{800}$ = 0.25

1. Kg Mukut ( 2° 43’ 21” N, 104° 10’ 42”E)

- Area = ( 0.6 × 2) + ( 1 × 1.9) + ( 1.6 × 0.6)

= 1.2 + 1.9 + 0.96

= 4.06cm^2^

= 1.015km^2^

- Community hall, clinic, post office, mosque, postal agency, wireless mast
- Vegetation:

1. Secondary jungle, Belukar, Riung and Jerami
2. Coconut
3. S. Raya

- Highest elevation: 820m
- Possible intake : 140m ( 2° 43’ 45” N, 104° 10’ 39”E)
- Catchment area = (1.1 ×4) + ( 2.3 × 5.3) + ( 0.7 × 3.9) + ( 0.8 × 0.5)
- = 4.4 + 12.19 + 2.73 + 1.3

= 20.62cm^2^

= 5.2km^2^

- Single line river
- Tailrace= 60m
- Available head = 140-60

= 80m

- River gradient = $\frac{140-15}{1.7cm \times\frac{1km}{2cm}\times\frac{1000 m}{1 km}}$ =$\frac{125}{850}$ = 0.147

1. S. Air Demit

- There are buildings located alongside the river up to 100m
- Highest elevation: 340m
- Possible intake : 120m ( 2° 43’ 37” N, 104° 11’ 18”E)
- Catchment area = (1.3 ×1)

= 1.3cm^2^

= 0.325km^2^

- Single line river
- Tailrace= 100m
- Available head = 120-100

= 20m

- River gradient = $\frac{120-15}{1cm \times\frac{1km}{2cm}\times\frac{1000 m}{1 km}}$ =$\frac{105}{500}$ = 0.21

1. Kg Asah ( 2° 43’ 15” N, 104° 11’ 40”E)

- Small energy demand, few buildings
- Area = ( 0.7 × 0.7)

= 0.49cm^2^

= 0.1225km^2^

- Vegetation:

1. Secondary jungle, Belukar, Riung and Jerami
2. Sundry tree cultivation
3. S. Asah

- Highest elevation: 600m
- Possible intake : 160m ( 2° 43’ 41” N, 104° 11’ 35”E)
- Catchment area = (0.3 ×1.2) + ( 1.1 × 0.5) + ( 1.9 × 0.5) + ( 1.5 × 3.2) + ( 1.2 × 2.2)

= 0.36 + 0.55+ 0.95 + 4.8 + 2.64

= 9.3cm^2^

= 2.33km^2^

- Single line river
- Tailrace= 20m
- Available head = 160-20

= 140m

- River gradient = $\frac{160}{1.6cm \times\frac{1km}{2cm}\times\frac{1000 m}{1 km}}$ =$\frac{160}{800}$ = 0.2
- Got waterfall at 80m

1. S. Kelayuk

- Highest elevation: 360m
- Possible intake : 220m ( 2° 43’ 32” N, 104° 12’ 09”E)
- Catchment area = (0.6 ×0.6)

= 0.36cm^2^

= 0.09km^2^

- Single line river
- Tailrace= 20m
- Available head = 220-20

= 200m

- River gradient = $\frac{220}{1.4cm \times\frac{1km}{2cm}\times\frac{1000 m}{1 km}}$ =$\frac{220}{700}$ = 0.314

1. Tg Benuang ( 2° 44’ 21” N, 104° 13’ 15”E)

- Area = NA
- Vegetation:

1. Secondary jungle, Belukar, Riung and Jerami
2. Coconut
3. Sundry tree cultivation
4. S. Benuang

- Highest elevation: 600m
- Possible intake : 160m ( 2° 44’ 29” N, 104° 12’ 32”E)
- Catchment area = (2.4 ×2.8)

= 6.77cm^2^

= 1.68km^2^

- Single line river
- Tailrace= 20m
- Available head = 160-20

= 140m

- River gradient = $\frac{160}{1.7cm \times\frac{1km}{2cm}\times\frac{1000 m}{1 km}}$ =$\frac{160}{850}$ = 0.188

1. Tg Niting ( 2° 45’ 23” N, 104° 13’ 20”E)

- Area = NA
- Vegetation:

1. Secondary jungle, Belukar, Riung and Jerami
2. Coconut
3. Sundry tree cultivation
4. S. Air Seler

- Highest elevation: 800m
- Possible intake : 2000m ( 2° 45’ 26” N, 104° 12’ 29”E)
- Catchment area = (3.3 ×4.2)

= 13.86cm^2^

= 3.465km^2^

- Single line river
- Tailrace= 20m
- Available head = 200-20

= 180m

- River gradient = $\frac{200}{3cm \times\frac{1km}{2cm}\times\frac{1000 m}{1 km}}$ =$\frac{200}{1500}$ = 0.133

1. Kg Juara ( 2° 47’ 41” N, 104° 12’ 11”E)

- Community hall, mosque, 2 wooden bridge,
- Area = ( 1.3 × 0.7) + ( 1.7 × 1.1) + ( 2.2 × 1) + ( 1 × 0.8) + ( 0.8 × 1)

= 0.91 + 1.87 + 2.2 + 0.8 + 0.88

= 6.66cm^2^

= 1.67km^2^

- Vegetation:

1. Secondary jungle, Belukar, Riung and Jerami
2. Coconut
3. Sundry tree cultivation
4. S. Mentawak

- Highest elevation: 1020m
- Possible intake : 160m ( 2° 46’ 31” N, 104° 11’ 10”E)
- Catchment area = (0.9 ×4.2) + ( 0.6 × 5.2) + ( 2.3 × 5.9) + ( 3.8 × 7.2) + ( 0.5 × 5.6) + ( 0.3 × 4.4)
- = 3.78 + 3.12+ 3.57 + 27.36 + 2.8 + 1.32

= 51.95cm^2^

= 13km^2^

- Single line river
- Tailrace= 20m
- Available head = 160-20

= 140m

- River gradient = $\frac{160}{4cm \times\frac{1km}{2cm}\times\frac{1000 m}{1 km}}$ =$\frac{160}{2000}$ = 0.08
- Biggest river in Tioman island
- There are more than one possible intake for S Mentawak up to 240m. Since it is a big river with a lot of tributaries and confluence. There can be more than one hydropower scheme for S Mentawak. This study only consider the best possible option for hydropower generation.

1. S. Keliling

- Highest elevation: 860m
- Possible intake : 200m ( 2° 47’ 34” N, 104° 11’ 15”E)
- Catchment area = (2.1 ×4.2) + ( 0.7 × 1.3)
- = 8.82 + 0.91

= 9.73cm^2^

= 2.4km^2^

- Single line river
- Tailrace= 10m
- Available head = 200-10

= 190m

- River gradient = $\frac{200-10}{2.6cm \times\frac{1km}{2cm}\times\frac{1000 m}{1 km}}$ =$\frac{190}{20001300}$ = 0.15

1. S. Baharu

- Highest elevation: 660m
- Possible intake : 160m ( 2° 48’ 11” N, 104° 11’ 21”E)
- Catchment area = (0.2 ×0.8) + ( 0.15 × 1.3) + ( 3.2 × 3.1)

= 0.16 + 0.195+ 9.92

= 10.275cm^2^

= 2.57km^2^

- Single line river
- Tailrace= 20m
- Available head = 160-20

= 140m

- River gradient = $\frac{160-10}{3.1cm \times\frac{1km}{2cm}\times\frac{1000 m}{1 km}}$ =$\frac{150}{1550}$ = 0.097

- S. Sabut, S. Air Dalam, S. Air Keruh- no hydro potential

- S. Sabut, S. Air Dalam, S. Baharu flows into the same river stem.

- S. Sabut < 60m head and small catchment area

- S. Air Dalam, S. Air Keruh no hydro potential since there are no tributaries and confluence.

1. Kg Dungun ( 2° 49’ 55” N, 104° 11’ 39”E)

- Area = NA
- Vegetation:

1. Secondary jungle, Belukar, Riung and Jerami
2. Coconut
3. Sundry tree cultivation
4. S. Dungun

- Highest elevation: 440m
- Possible intake : 160m ( 2° 49’ 54” N, 104° 11’ 06”E)
- Catchment area = (2.2 ×2) + (0.55 ×1)

= 4 + 0.55

= 4.55cm^2^

= 1.14km^2^

- Single line river
- Tailrace= 20m
- Available head = 160-20

= 140m

- River gradient = $\frac{160}{2.3cm \times\frac{1km}{2cm}\times\frac{1000 m}{1 km}}$ =$\frac{160}{1150}$ = 0.1391

1. Tk Tarah ( 2° 51’ 52” N, 104° 11’ 10”E)

- Area = NA
- Vegetation:

1. Secondary jungle, Belukar, Riung and Jerami
2. Coconut
3. Sundry tree cultivation
4. S. Tarah

- Highest elevation: 500m
- Possible intake : 150m ( 2° 51’ 52” N, 104° 10’ 39”E)
- Catchment area = (2.4 ×2.3)

= 5.52cm^2^

= 1.38km^2^

- Single line river
- Tailrace= 20m
- Available head = 150-20

= 130m

- River gradient = $\frac{150}{1.9cm \times\frac{1km}{2cm}\times\frac{1000 m}{1 km}}$ =$\frac{150}{950}$ = 0.16

1. Kg Teluk Salang ( 2° 52’ 44” N, 104° 09’ 29”E)

- Community hall
- Area = ( 2.5 × 1) + ( 0.6 × 0.5)

= 2.5 + 0.3

= 2.8cm^2^

= 0.7km^2^

- Vegetation:

1. Secondary jungle, Belukar, Riung and Jerami
2. Sundry tree cultivation
3. S. Salang

- No Hydro potential since available head < 20m

1. Kg Penuba ( 2° 51’ 06” N, 104° 09’ 28”E)

- No river
- No hydo
- Near to kg Air Batang
- Area = ( 0.8 × 1) + ( 0.3 × 0.4)

= 0.8 + 0.12

= 0.92cm^2^

= 0.23km^2^

- Vegetation:

1. Coconut
2. Kg Air Batang ( 2° 50’ 38” N, 104° 09’ 49”E)

- 1 lighthouse/ lighted beacon/lighted buoy, 1 water tank, tg mesoh
- Area = ( 3.1 × 1.1)

= 3.41cm^2^

= 0.85km^2^

- Vegetation:

1. Coconut
2. S. Air Batang

- Highest elevation: 420m
- Possible intake : 240m ( 2° 50’ 57” N, 104° 10’ 18”E)
- Catchment area = (0.9 ×1.1)

= 0.99cm^2^

= 0.248km^2^

- Single line river
- Tailrace= 100m
- Available head = 240-100

= 140m

- River gradient = $\frac{240}{2.4cm \times\frac{1km}{2cm}\times\frac{1000 m}{1 km}}$ =$\frac{240}{1200}$ = 0.2
- In between kg Penuba and kg Air Batang

Analysis for Tioman island

There are 18 location has been studied for hydropower potential assessment in Tioman island

1. Pulau Tioman

| No | Location | No. of Potential Sites |
| --- | --- | --- |
|  | Kg Tekek | 4 |
|  | Kg Lalang | 1 |
|  | Kg Bunut | 1 |
|  | Kg Paya | 1 |
|  | Kg Genting | 3 |
|  | Tg Permata | 0 |
|  | Tg K Nipah | 3 |
|  | Kg Pasir Sarang Burung | 1 |
|  | Kg Mukut | 2 |
|  | Kg Asah | 2 |
|  | Tg Benuang | 1 |
|  | Tg Niting | 1 |
|  | Kg Juara | 3 |
|  | Kg Dungun | 1 |
|  | Tk Tarah | 1 |
|  | Kg Teluk salang | 0 |
|  | Kg Penuba | 0 |
|  | Kg Air Batang | 1 |

| No | Location | Stream name | Available  head , m | Catchment area,km^2^ | River gradient |
| --- | --- | --- | --- | --- | --- |
|  | Kg Tekek | S Tekek | 180 | 0.303 | 0.168 |
|  |  | S Air Besar | 240 | 1.906 | 0.175 |
|  |  | S Batang Sabut | 100 | 0.375 | 0.147 |
|  |  | S Tg said | 200 | 0.0963 | 0.24 |
|  | Kg Lalang | S Lalang | 120 | 1.19 | 0.15 |
|  | Kg Bunut | S Setup | 180 | 0.618 | 0.271 |
|  | Kg Paya | S Paya | 220 | 2.4 | 0.1 |
|  | Kg Genting | S Air Jerangau | 180 | 0.1225 | 0.33 |
|  |  | S Air Raja | 120 | 1.67 | 0.16 |
|  |  | S Air Gong | 140 | 0.728 | 0.21 |
|  | Tg Permata | - | - | - | - |
|  | Tg K Nipah | S Kasut | 140 | 0.27 | 0.33 |
|  |  | S Nipah | 170 | 3.14 | 0.17 |
|  |  | S Seriah | 100 | 4.66 | 0.1 |
|  | Kg Pasir Sarang Burung | S Tedau | 180 | 1.05 | 0.25 |
|  | Kg Mukut | S Raya | 80 | 5.2 | 0.147 |
|  |  | S Air Demit | 20 | 0.325 | 0.21 |
|  | Kg Asah | S Asah | 140 | 9.3 | 0.2 |
|  |  | S Kelayuk | 200 | 0.09 | 0.314 |
|  | Tg Benuang | S Benuang | 140 | 1.68 | 0.188 |
|  | Tg Niting | S Air Seler | 180 | 3.465 | 0.133 |
|  | Kg Juara | S Mentawak | 140 | 13 | 0.08 |
|  |  | S Keliling | 180 | 2.4 | 0.15 |
|  |  | S Baharu | 140 | 2.57 | 0.097 |
|  | Kg. Dungun | S Dungun | 140 | 1.14 | 0.1319 |
|  | Tk. Tarah | S Tarah | 130 | 1.38 | 0.16 |
|  | Kg Teluk Salang | - | - | - | - |
|  | Kg Penuba | - | - | - | - |
|  | Kg Air Batang | S Air Batang | 140 | 0.248 | 0.2 |

1. **Pulau Perhentian**
2. **Pulau Perhentian Besar**

There are no hydro potential in pulau perhentian besar and very small energy demand

Rest house: Area= 0.7 ×0.5 = 0.35cm^2^ = 0.0875cm^2^

1. **Pulau Perhentian Kecil**

- Highest elevation (345m) - Trigonometry station

1. Kg Pasir Hantu ( 5° 53’ 52” N, 102° 43’ 52”E)

- Mosque, school, postal agency, clinic
- Area = ( 0.8 × 0.8)

= 0.64cm^2^

= 0.16km^2^

- Vegetation:

1. Secondary jungle, Belukar, Riung and Jerami
2. Rubber
3. Coconut
4. Sundry tree cultivation
5. Unnamed stream(Tk. Batu Jalil)

- Highest elevation: 280m
- Possible intake : 80m ( 5° 54’ 42” N, 102° 43’ 40”E)
- Catchment area = 1 ×0.8

= 0.8cm^2^

= 0.2km^2^

- Single line river
- Tailrace= 20m
- Available head = 80-20

= 60m

- River gradient = $\frac{80}{0.6cm \times\frac{1km}{2cm}\times\frac{1000 m}{1 km}}$ =$\frac{80}{300}$ = 0.27

1. Kg Pasir Petani ( 5° 53’ 46” N, 102° 43’ 20”E)

- Area = ( 0.4 × 0.4)

= 0.16cm^2^

= 0.04km^2^

- Vegetation:

1. Secondary jungle, Belukar, Riung and Jerami
2. Sundry tree cultivation
3. Unnamed stream (Pasir Karang)

- Highest elevation: 300m
- Possible intake : 100m ( 5° 54’ 15” N, 102° 43’ 16”E)
- Catchment area = 0.6 ×1.2

= 0.72cm^2^

= 0.18km^2^

- Single line river
- Tailrace= 20m
- Available head = 100-20

= 80m

- River gradient = $\frac{100}{0.9cm \times\frac{1km}{2cm}\times\frac{1000 m}{1 km}}$ =$\frac{100}{450}$ = 0.22

Analysis for Perhentian Kecil island

There are 2 location has been studied for hydropower potential assessment in Perhentian Kecil island

| No | Location | No. of Potential Sites |
| --- | --- | --- |
|  | Kg Pasir Hantu | 1 |
|  | Kg Pasir Petani | 1 |

| No | Location | Stream name | Available  head , m | Catchment area,km^2^ | River gradient |
| --- | --- | --- | --- | --- | --- |
|  | Kg Pasir Hantu | Unnamed  (Tk. Batu Jalil) | 60 | 0.2 | 0.27 |
|  | Kg Pasir Petani | Unnamed  (Pasir Karang) | 80 | 0.18 | 0.22 |

1. **Pulau Redang**

Mountains and Hills.

- Besar Hill (359m)
- Batu Kelinang Hill (240m)
- Telaga Batu Hill (229m)
- Kuala Hill (218m)
- Mat Sayang Hill (295m)

1. K Redang ( 5° 45’ 23” N, 103° 00’ 24”E)

- School, clinic, postal agency, mosque
- Area = ( 1.4 × 0.7)

= 0.98cm^2^

= 0.245km^2^

- Vegetation:

1. Rubber
2. Sundry tree cultivation
3. Unnamed stream (Tg. Telaga Batu)

- Highest elevation: 180m
- Possible intake : 80m ( 5° 45’ 11” N, 103° 00’ 47”E)
- Catchment area = 0.8 × 0.7

= 0.56cm^2^

= 0.14km^2^

- Single line river
- Tailrace= 20m
- Available head = 80-20

= 60m

- River gradient = $\frac{80}{0.5cm \times\frac{1km}{2cm}\times\frac{1000 m}{1 km}}$ =$\frac{80}{250}$ = 0.32

1. Kg. Tk. Bakau ( 5° 45’ 51” N, 103° 01’ 37”E)

- Agriculture office (pejabat pertanian)
- Area = ( 0.45 × 0.9)

= 0.405cm^2^

= 0.101km^2^

- Vegetation:

1. Belukar, Riung and Hill padi
2. Coconut
3. Unnamed stream (Tk. Kalung Besar)

- Highest elevation: 180m
- Possible intake : 100m ( 5° 45’ 41” N, 103° 01’ 12”E)
- Catchment area = 0.7 × 0.8

= 0.56cm^2^

= 0.14km^2^

- Single line river
- Tailrace= 20m
- Available head = 100-20

= 80m

- River gradient = $\frac{100}{1.2cm \times\frac{1km}{2cm}\times\frac{1000 m}{1 km}}$ =$\frac{100}{600}$ = 0.167

1. Kg. Tk. Dalam ( 5° 47’ 07” N, 103° 01’ 02”E)

- Rest house
- Area = ( 0.4 × 0.4) + ( 0.4 × 0.7)

= 0.16 + 0.28

= 0.44cm^2^

= 0.11km^2^

- Vegetation:

1. Coconut
2. Unnamed stream (Tk. Dalam)

- Highest elevation: 160m
- Possible intake : 110m ( 5° 46’ 41” N, 103° 01’ 21”E)
- Catchment area = 0.5 × 0.7

= 0.35cm^2^

= 0.0875km^2^

- Single line river
- Tailrace= 20m
- Available head = 110-20

= 90m

- River gradient = $\frac{110}{1.6cm \times\frac{1km}{2cm}\times\frac{1000 m}{1 km}}$ =$\frac{110}{800}$ = 0.138

Analysis for Pulau Redang

There are 3 location has been studied for hydropower potential assessment in Redang island

| No | Location | No. of Potential Sites |
| --- | --- | --- |
|  | K Redang | 1 |
|  | Kg Tk. Bakau | 1 |
|  | Kg. Tk. Dalam | 1 |

| No | Location | Stream name | Available  head , m | Catchment area,km^2^ | River gradient |
| --- | --- | --- | --- | --- | --- |
|  | K Redang | Unnamed  (Tg. Telaga Batu) | 60 | 0.14 | 0.32 |
|  | Kg Tk. Bakau | Unnamed  (Tk. Kalung Besar) | 80 | 0.14 | 0.167 |
|  | Kg. Tk. Dalam | Unnamed  (Tk. Dalam) | 90 | 0.0875 | 0.138 |

1. **Pulau Lang Tengah**

- No hydro potential
- Vegetation:

1. Belukar, Riung and Hill Padi
2. Coconut

- Highest elevation is 128m

1. **Pulau Tenggol**

- No hydro potential
- No river/stream
- Highest elevation is 253m
- Lighthouse
- Small energy demand based on topographic map.
- No infrastructures / buildings.

1. **Pulau Gemia**

- Identified as Pulau gumia(in topo map)
- Very small island
- Gem island resort
- No hydro potential
- Highest elevation is 45m

1. **Pulau Kapas**
2. Pulau Kerengga ( 05° 13’ 06” N, 103° 15’ 48”E)

- Area = ( 0.8 × 0.5) + ( 0.4 × 0.4)

= 0.4 + 0.16

= 0.56cm^2^

= 0.14km^2^

- Vegetation:

1. Coconut
2. Tk. Berakit

- Highest elevation: 60m
- Possible intake : 25m ( 5° 13’ 30” N, 103° 15’ 03”E)
- Catchment area = 0.4 ×0.4

= 0.16cm^2^

= 0.04km^2^

- Single line river
- Tailrace= 10m
- Available head = 25-10

= 15m

- River gradient = $\frac{25}{0.7cm \times\frac{1km}{2cm}\times\frac{1000 m}{1 km}}$ =$\frac{25}{350}$ = 0.0714
- Difficult for transmission because of hilly regions and distance
- Not recommended site for hydropower development

Analysis for Pulau Kapas

There is 1 location has been studied for hydropower potential assessment in Perhentian Kapas

| No | Location | No. of Potential Sites |
| --- | --- | --- |
|  | Pulau Kerengga | 1 |

| No | Location | Stream name | Available  head , m | Catchment area,km^2^ | River gradient |
| --- | --- | --- | --- | --- | --- |
|  | Pulau Kerengga | Tk. Berakit | 15 | 0.04 | 0.0714 |

1. **Pulau Aur**

Mountains and Hills.

- Belakang Parang Hill (421m)
- Bt. Makum (521m) –Trigonometrical station

1. Kg. Tk. Berhala ( 2° 27’ 50” N, 104° 30’ 28”E)

- Police station, community hall, school, clinic, postal agency
- Calet Tk. Bail, Calet Tk. Berhala
- Area = ( 1.4 × 1.5) + ( 0.5 × 0.5)

= 2.1 + 0.25

= 2.35cm^2^

= 0.5875km^2^

- Vegetation:

1. Coconut
2. S. Berhala

- Highest elevation: 280m
- Possible intake : 220m ( 2° 27’ 42” N, 104° 30’ 52”E)
- Catchment area = 1 × 0.8

= 0.8cm^2^

= 0.2km^2^

- Single line river
- Tailrace= 20m
- Available head = 220-40

= 180m

- River gradient = $\frac{220}{2.5cm \times\frac{1km}{2cm}\times\frac{1000 m}{1 km}}$ =$\frac{220}{1250}$ = 0.176

1. Kg. Sabut ( 2° 27’ 11” N, 104° 30’ 18”E)

- Calet Tk. Minyang
- Area = ( 1.3 × 0.5)

= 0.65cm^2^

= 0.1625km^2^

- Vegetation:

1. Coconut
2. Tk. Minyang

- Highest elevation: 320m
- Possible intake : 265m ( 2° 27’ 19” N, 104° 30’ 32”E)
- Catchment area = 0.7 × 0.9

= 0.63cm^2^

= 0.158km^2^

- Single line river
- Tailrace= 40m
- Available head = 265-40

= 225m

- River gradient = $\frac{265}{1.1cm \times\frac{1km}{2cm}\times\frac{1000 m}{1 km}}$ =$\frac{265}{550}$ = 0.482

1. Kg. Tk. Jong ( 2° 26’ 50” N, 104° 30’ 21”E)

- Area = ( 1.2 × 0.6) + ( 0.3 × 1.6)

= 0.72 + 0.48

= 1.2cm^2^

= 0.3km^2^

- Vegetation:

1. Coconut
2. S. Putat/ Tg. Pusong

- Highest elevation: 340m
- Possible intake : 220m ( 2° 26’ 57” N, 104° 30’ 52”E)
- Catchment area = 1 × 0.7

= 0.7cm^2^

= 0.175km^2^

- Single line river
- Tailrace= 20m
- Available head = 220-20

= 200m

- River gradient = $\frac{220}{1.4cm \times\frac{1km}{2cm}\times\frac{1000 m}{1 km}}$ =$\frac{220}{700}$ = 0.314

1. Kg. Tk. Meriam ( 2° 26’ 31” N, 104° 30’ 54”E)

- Area = ( 0.6 × 0.3) + ( 0.2 × 0.2) + ( 0.3 × 0.6)

= 0.18 + 0.04 + 0.18

= 0.4cm^2^

= 0.1km^2^

- Vegetation:

1. Coconut
2. S. Kepayang

- Highest elevation: 220m
- Possible intake : 160m ( 2° 26’ 47” N, 104° 31’ 06”E)
- Catchment area = 0.55 × 1.1

= 0.605cm^2^

= 0.151km^2^

- Single line river
- Tailrace= 20m
- Available head = 160-20

= 140m

- River gradient = $\frac{160}{1.2cm \times\frac{1km}{2cm}\times\frac{1000 m}{1 km}}$ =$\frac{160}{600}$ = 0.267

1. S. Galing

- Highest elevation: 440m
- Possible intake : 120m ( 2° 26’ 24” N, 104° 31’ 24”E)
- Catchment area = (1.2 × 1.5) + (0.5 × 0.7)

= 1.8 + 0.35

= 2.15cm^2^

= 0.5375km^2^

- Single line river
- Tailrace= 20m
- Available head = 120-20

= 100m

- River gradient = $\frac{120}{2cm \times\frac{1km}{2cm}\times\frac{1000 m}{1 km}}$ =$\frac{120}{1000}$ = 0.12

1. Kg. Palas ( 2° 25’ 36” N, 104° 31’ 38”E)

- Area = ( 0.4 × 0.5)

= 0.2cm^2^

= 0.05km^2^

- Vegetation:

1. Coconut
2. S. Kerasan

- Highest elevation: 380m
- Possible intake : 100m ( 2° 25’ 50” N, 104° 31’ 28”E)
- Catchment area = (0.75 × 0.7) + (1.1 × 0.7)

= 0.525 + 0.77

= 1.295cm^2^

= 0.324km^2^

- Single line river
- Tailrace= 20m
- Available head = 100-20

= 80m

- River gradient = $\frac{100}{0.9cm \times\frac{1km}{2cm}\times\frac{1000 m}{1 km}}$ =$\frac{100}{480}$ = 0.22

1. Kg. Teluran ( 2° 27’ 28” N, 104° 31’ 37”E)

- Area = ( 1 × 0.5)

= 0.5cm^2^

= 0.125km^2^

- Vegetation:

1. Coconut

-S Terantang- no hydro potential, available head <20m

1. S. Teluran

- Highest elevation: 360m
- Possible intake : 100m ( 2° 27’ 14” N, 104° 31’ 19”E)
- Catchment area = (0.75 × 0.7) + (1.1 × 0.7)

= 1.4 + 1.4

= 1.96cm^2^

= 0.49km^2^

- Single line river
- Tailrace= 20m
- Available head = 100-20

= 80m

- River gradient = $\frac{100}{1.7cm \times\frac{1km}{2cm}\times\frac{1000 m}{1 km}}$ =$\frac{100}{850}$ = 0.118

1. Kg. Kelabu ( 2° 28’ 00” N, 104° 31’ 00”E)

-S Berhala- no hydro potential

Analysis for Aur island

There are 7 location has been studied for hydropower potential assessment in Aur island

| No | Location | No. of Potential Sites |
| --- | --- | --- |
|  | Kg. Tk. Berhala | 1 |
|  | Kg. Sabut | 1 |
|  | Kg. Tk. Jong | 1 |
|  | Kg. Tk. Meriam | 2 |
|  | Kg. Palas | 1 |
|  | Kg. Teluran | 1 |
|  | Kg. Kelabu | 0 |

| No | Location | Stream name | Available  head , m | Catchment area,km^2^ | River gradient |
| --- | --- | --- | --- | --- | --- |
|  | Kg. Tk. Berhala | S. Berhala | 180 | 0.2 | 0.176 |
|  | Kg. Sabut | Tk. Minyang | 225 | 0.158 | 0.482 |
|  | Kg. Tk. Jong | S. Putat | 200 | 0.175 | 0.314 |
|  | Kg. Tk. Meriam | S. Kepayang | 140 | 0.151 | 0.267 |
|  |  | S. Galing | 100 | 0.5375 | 0.12 |
|  | Kg. Palas | S. Kerasan | 80 | 0.324 | 0.22 |
|  | Kg. Teluran | S. Teluran | 80 | 0.49 | 0.48 |
|  | Kg. Kelabu | - | - | - | - |

1. **Pulau Tinggi**

Mountains and Hills.

- P. Tinggi (610m) - Trigonometrical station
- Ulu Terih Hill(281m)
- Semuntok Hill (308m)
- Semundu Kechil Hill( 315m)

1. Kg. Buloh Kasap ( 2° 18’ 48” N, 104° 06’ 02”E)

- Area = ( 0.4 × 0.3)

= 0.12cm^2^

= 0.03km^2^

- Vegetation:

1. Coconut
2. Secondary jungle, belukar, riung and jerami
3. S. Buloh Kasap

- Highest elevation: 200m
- Possible intake : 100m ( 2° 18’ 23” N, 104° 06’ 24”E)
- Catchment area = 0.5 × 0.4

= 0.2cm^2^

= 0.05km^2^

- Single line river
- Tailrace= 20m
- Available head = 100-20

= 80m

- River gradient = $\frac{100}{1.4cm \times\frac{1km}{2cm}\times\frac{1000 m}{1 km}}$ =$\frac{100}{700}$ = 0.143

1. Kg. Sebirah Besar ( 2° 18’ 41” N, 104° 05’ 52”E)

- School
- Area = ( 1 × 0.4)

= 0.4cm^2^

= 0.1km^2^

- Vegetation:
  1. Secondary jungle, Belukar, Riung and Jerami
  2. Rubber
- There are no hydropower potential in Kg. Sebirah Besar. Since S. Buloh Kasap is the middle of Kg. Buloh Kasap and Kg. Sebirah Besar. It can be considered for hydropower source in this village.

1. Kg. Sebirah Kecil( 2° 18’ 15” N, 104° 05’ 52”E)

- Area = ( 0.9 × 0.6)

= 0.54cm^2^

= 0.135km^2^

- Vegetation:

1. Secondary jungle, Belukar, Riung and Jerami
2. Rubber

- There are no hydropower potential in Kg. Sebirah Kasap. Since S. Terih Kecil is near Kg. Kg. Sebirah Kecil. It can be considered for hydropower source for the village.

1. Kg. Tk. Sauk ( 2° 17’ 58” N, 104° 06’ 04”E)

- Area = ( 0.8 × 1.1)

= 0.88cm^2^

= 0.22km^2^

- Vegetation:

1. Secondary jungle, Belukar, Riung and Jerami
2. Rubber
3. Kg. Pasir Panjang ( 2° 17’ 42” N, 104° 06’ 13”E)

- Area = ( 0.6 × 1.3)

= 0.78cm^2^

= 0.195km^2^

- Vegetation:

1. Secondary jungle, Belukar, Riung and Jerami
2. Rubber
3. Kg. Terih ( 2° 17’ 31” N, 104° 06’ 39”E)

- Area = ( 0.4 × 0.6)

= 0.24cm^2^

= 0.06km^2^

- Vegetation:

1. Secondary jungle, Belukar, Riung and Jerami
2. Rubber
3. Kg. Tanah Tumboh ( 2° 17’ 37” N, 104° 06’ 31”E)

- Area = ( 0.7 × 0.7) + ( 0.3 × 0.4)

= 0.49 + 0.12

= 0.61cm^2^

= 0.153km^2^

- Vegetation:

1. Coconut
2. Secondary jungle, belukar, riung and jerami
3. S. Terih Kechil

- Highest elevation: 420
- Possible intake : 140m ( 2° 18’ 02” N, 104° 06’ 39”E)
- Catchment area = 1.3 × 1.5

= 1.95cm^2^

= 0.488km^2^

- Single line river
- Tailrace= 20m
- Available head = 140-20

= 120m

- River gradient = $\frac{140}{2.2cm \times\frac{1km}{2cm}\times\frac{1000 m}{1 km}}$ =$\frac{140}{1100}$ = 0.127
- S. Terih is near to Kg, Tk. Sauk, Kg. Parang Panjang, Kg. Tanah Tumboh and Kg. Terih

1. Kg. Kelopak ( 2° 17’ 10” N, 104° 06’ 57”E)

- Area = ( 0.4 × 0.7)

= 0.28cm^2^

= 0.07km^2^

- Vegetation:

1. Coconut
2. Secondary jungle, belukar, riung and jerami
3. S. Terih Besar

- Highest elevation: 400
- Possible intake : 80m ( 2° 17’ 41” N, 104° 07’ 07”E)
- Catchment area = (0.3 × 0.6) + (0.8 × 1.3) +(0.55 × 0.8) +(0.4 × 0.4)

= 0.18 + 1.04 + 0.44 + 0.16

= 1.82cm^2^

= 0.455km^2^

- Single line river
- Tailrace= 20m
- Available head = 80-20

= 60m

- River gradient = $\frac{80}{2.1cm \times\frac{1km}{2cm}\times\frac{1000 m}{1 km}}$ =$\frac{80}{1050}$ = 0.0762

1. Kg.Penaga ( 2° 17’ 10” N, 104° 07’ 15”E)

- Area = ( 0.6 × 1.2)

= 0.72cm^2^

= 0.18km^2^

- Vegetation:

1. Coconut
2. Secondary jungle, belukar, riung and jerami
3. Kg.Ayer Kolam ( 2° 17’ 18” N, 104° 07’ 19”E)

- Area = ( 0.5 × 0.6)

= 0.3^2^

= 0.075km^2^

- Vegetation:

1. Coconut
2. Secondary jungle, belukar, riung and jerami
3. Kg. Sulor Gading ( 2° 17’ 11” N, 104° 07’ 44”E)

- Area = ( 0.8 × 0.5)

= 0.4cm^2^

= 0.1km^2^

- Vegetation:

1. Coconut
2. Secondary jungle, belukar, riung and jerami
3. Kg. Kota ( 2° 17’ 02” N, 104° 07’ 45”E)

- Area = ( 0.5 × 0.5)

= 0.25cm^2^

= 0.0625km^2^

- Vegetation:

1. Coconut
2. Secondary jungle, belukar, riung and jerami
3. Kg. Tg. Balang ( 2° 17’ 02” N, 104° 07’ 21”E)

- Mosque, postal agency, shool, clinic
- Area = ( 0.6× 1.1)

= 0.66cm^2^

= 0.165km^2^

- Vegetation:

1. Coconut
2. Secondary jungle, belukar, riung and jerami

- S. Terih Besar is near to Kg Penaga, Kg. Ayer Kolam, Kg. Sulor Gading, Kg. Kota and Kg. Tg. Balang.

1. Kg. Tk. Pinang ( 2° 17’ 26” N, 104° 08’ 13”E)

- Area = ( 0.7 × 0.6)

= 0.42cm^2^

= 0.105km^2^

- Vegetation:

1. Coconut
2. Secondary jungle, belukar, riung and jerami
3. S. Tk. Pinang

- Highest elevation: 440
- Possible intake : 80m ( 2° 17’ 50” N, 104° 07’ 39”E)
- Catchment area = (1.1 × 1.4)

= 1.54cm^2^

= 0.385km^2^

- Single line river
- Tailrace= 20m
- Available head = 80-20

= 60m

- River gradient = $\frac{80}{3cm \times\frac{1km}{2cm}\times\frac{1000 m}{1 km}}$ =$\frac{80}{1500}$ = 0.0533
- There are potential for higher head but smaller catchment area.

Analysis for Tinggi island

There are 14 location has been studied for hydropower potential assessment in Tinggi island

| No | Location | No. of Potential Sites |
| --- | --- | --- |
|  | Kg. Buloh Kasap | 1 |
|  | Kg. Sebirah Besar | 0 |
|  | Kg. Sebirah Kecil | 0 |
|  | Kg. Tk. Sauk | 0 |
|  | Kg. Pasir Panjang | 0 |
|  | Kg. Terih | 0 |
|  | Kg. Tanah Tumboh | 1 |
|  | Kg. Kelopak | 1 |
|  | Kg. Penaga | 0 |
|  | Kg. Ayer Kolam | 0 |
|  | Kg. Sulor Gading | 0 |
|  | Kg. Kota | 0 |
|  | Kg. Tg. Balang | 0 |
|  | Kg. Tk. Pinang | 1 |

| No | Location | Stream name | Available  head , m | Catchment area,km^2^ | River gradient |
| --- | --- | --- | --- | --- | --- |
|  | Kg. Buloh Kasap | S. Buloh Kasap | 80 | 0.05 | 0.143 |
|  | Kg. Sebirah Besar | - | - | - | - |
|  | Kg. Sebirah Kecil | - | - | - | - |
|  | Kg. Tk. Sauk | - | - | - | - |
|  | Kg. Pasir Panjang | - | - | - | - |
|  | Kg. Terih | - | - | - | - |
|  | Kg. Tanah Tumboh | S. Terih Kecil | 120 | 0.488 | 0.127 |
|  | Kg. Kelopak | S. Terih Besar | 60 | 0.455 | 0.0762 |
|  | Kg. Penaga | - | - | - | - |
|  | Kg. Ayer Kolam | - | - | - | - |
|  | Kg. Sulor Gading | - | - | - | - |
|  | Kg. Kota | - | - | - | - |
|  | Kg. Tg. Balang | - | - | - | - |
|  | Kg. Tk. Pinang | S. Tk. Pinang | 60 | 0.385 | 0.0533 |

1. **Pulau Sibu**

Mountains and Hills.

- Sibu (155m)

1. Kg. Duku ( 2° 12’ 15” N, 104° 04’ 37”E)

- School, clinic, mosque
- Area = ( 1.1 × 0.6) + ( 0.4 × 0.3) + ( 0.5 × 0.5) +( 0.4 × 0.8) + ( 0.4 × 0.5)

= 0.66 + 0.12 + 0.25 + 0.32 + 0.2

= 1.55cm^2^

= 0.388km^2^

- Vegetation:

1. coconut
2. S. Duku

- Highest elevation: 120m
- Possible intake : 40m ( 2° 12’ 16” N, 104° 04’ 54”E)
- Catchment area = 0.5 × 0.5

= 0.25cm^2^

= 0.0625km^2^

- Single line river
- Tailrace= 20m
- Available head = 40-20

= 20m

- River gradient = $\frac{40}{1.6cm \times\frac{1km}{2cm}\times\frac{1000 m}{1 km}}$ =$\frac{40}{800}$ = 0.05
- The river is not clear in the topo map, unable to determine the discharge wether at Tk. Tagar/ Tg. Busong

1. Kg. Lama ( 2° 12’ 45” N, 104° 04’ 39”E)

- Area = ( 0.8 × 0.5) + ( 0.6 × 0.8)

= 0.4 + 0.48

= 0.88cm^2^

= 0.22km^2^

- Vegetation:

1. Coconut
2. Kg. Kambau ( 2° 13’ 30” N, 104° 04’ 00”E)

- Calet
- Area = ( 1 × 0.7)

= 0.7cm^2^

= 0.175km^2^

- Vegetation:

1. Coconut
2. Secondary jungle, Belukar, Riung and Jerami
3. Tg. Semanggar ( 2° 13’ 52” N, 104° 03’ 47”E)

- Calet
- Area = ( 0.4 × 0.3)

= 0.12cm^2^

= 0.03km^2^

- Vegetation:

1. Secondary jungle, Belukar, Riung and Jerami

Analysis for Sibu island

There are 4 location has been studied for hydropower potential assessment in Sibu island

| No | Location | No. of Potential Sites |
| --- | --- | --- |
|  | Kg. Duku | 1 |
|  | Kg. Lama | 0 |
|  | Kg. Kambau | 0 |
|  | Tg. Semanggar | 0 |

| No | Location | Stream name | Available  head , m | Catchment area,km^2^ | River gradient |
| --- | --- | --- | --- | --- | --- |
|  | Kg. Duku | S. Duku | 20 | 0.0625 | 0.05 |
|  | Kg. Lama | - | - | - | - |
|  | Kg. Kambau | - | - | - | - |
|  | Tg. Semanggar | - | - | - | - |

1. **Pulau Rawa**

- No hydropower potential at pulau rawa
- Small island with no hilly regions
- Highest elevation: 97m

1. **Pulau Pemanggil**

Mountains and Hills.

- Pontianak Hill (365m)
- Topo Hill (240m) – Trigonometrical station

1. Kg. Pontianak ( 2° 35’ 19” N, 104° 18’ 49”E)

- Area = ( 1.6 × 0.4)

= 0.64cm^2^

= 0.16km^2^

- Vegetation:

1. Coconut
2. Kg. Buau ( 2° 34’ 58” N, 104° 19’ 05”E)

- School, clinic, chalet, ,mosque, police station, postal agency, community hall
- Area = ( 0.4 × 0.6) + ( 0.9 × 0.5)

= 0.24 + 0.45

= 0.69cm^2^

= 0.1725km^2^

- Vegetation:

1. Coconut
2. Unnamed stream (Tk. Buau)

- Highest elevation: 300m
- Possible intake : 160m ( 2° 35’ 03” N, 104° 19’ 28”E)
- Catchment area = 0.7 × 0.45

= 0.315cm^2^

= 0.0788km^2^

- Single line river
- Tailrace= 20m
- Available head = 160-20

= 140m

- River gradient = $\frac{160}{1.4cm \times\frac{1km}{2cm}\times\frac{1000 m}{1 km}}$ =$\frac{160}{700}$ = 0.229

1. Kg. Sulit ( 2° 34’ 36” N, 104° 19’ 18”E)

- Area = ( 0.6 × 0.2) + ( 0.4 × 0.8)

= 0.12 + 0.32

= 0.44cm^2^

= 0.11km^2^

- Vegetation:

1. Coconut
2. Unnamed stream (Tk. Sulit)

- Highest elevation: 200m
- Possible intake : 130m ( 2° 34’ 30” N, 104° 19’ 32”E)
- Catchment area = 0.4 × 0.3

= 0.12cm^2^

= 0.03km^2^

- Single line river
- Tailrace= 20m
- Available head = 130-20

= 110m

- River gradient = $\frac{130}{1cm \times\frac{1km}{2cm}\times\frac{1000 m}{1 km}}$ =$\frac{130}{500}$ = 0.26
- Small river, low potential

1. Kg. Pa’ Saleh ( 2° 34’ 13” N, 104° 19’ 44”E)

- Area = ( 0.9 × 0.8) + ( 0.6 × 0.5) + ( 0.6 × 0.6)

= 0.72 + 0.3 + 0.36

= 1.38cm^2^

= 0.345km^2^

- Vegetation:

1. Coconut
2. Unnamed stream (Tk. Pa’ Saleh)

- Highest elevation: 340m
- Possible intake : 110m ( 2° 34’ 28” N, 104° 20’ 00”E)
- Catchment area = (0.4 × 0.1) + (0.4 × 0.6)

= 0.4 + 0.24

= 0.64cm^2^

= 0.16km^2^

- Single line river
- Tailrace= 40m
- Available head = 110-40

= 70m

- River gradient = $\frac{110}{1.3cm \times\frac{1km}{2cm}\times\frac{1000 m}{1 km}}$ =$\frac{110}{650}$ = 0.169

1. Tk. Lanting ( 2° 33’ 49” N, 104° 20’ 26”E)

- Area = ( 0.5 × 1.3)

= 0.65cm^2^

= 0.1625km^2^

- Vegetation:

1. Coconut

- No hydro potential

1. Tg. Sauk ( 2° 35’ 21” N, 104° 20’ 06”E)

- Area = ( 0.7 × 0.7)

= 0.49cm^2^

= 0.1225km^2^

- Vegetation:

1. Coconut
2. Unnamed stream (Tk. Sauk)

- Highest elevation: 260m
- Possible intake : 80m ( 2° 35’ 17” N, 104° 19’ 53”E)
- Catchment area = (0.9 × 1.1) + (0.3 × 0.7)

= 0.99 + 0.21

= 1.2cm^2^

= 0.3km^2^

- Single line river
- Tailrace= 40m
- Available head = 80-20

= 60m

- River gradient = $\frac{80}{1cm \times\frac{1km}{2cm}\times\frac{1000 m}{1 km}}$ =$\frac{80}{500}$ = 0.16

1. Tg. Lanchang( 2° 35’ 41” N, 104° 19’ 35”E)

- Area = ( 0.4 × 0.4)

= 0.16cm^2^

= 0.04km^2^

- Vegetation:

1. Coconut
2. Unnamed stream (Tk. Lanchang)

- Highest elevation: 260m
- Possible intake : 60m ( 2° 35’ 34” N, 104° 19’ 13”E)
- Catchment area = (0.5 × 0.5) + (0.7 × 0.5)

= 0.25 + 0.35

= 0.6cm^2^

= 0.15km^2^

- Single line river
- Tailrace= 20m
- Available head = 60-20

= 40m

- River gradient = $\frac{60}{1.8cm \times\frac{1km}{2cm}\times\frac{1000 m}{1 km}}$ =$\frac{60}{900}$ = 0.067

Analysis for Pemanggil island

There are 7 location has been studied for hydropower potential assessment in Pemanggil island

| No | Location | No. of Potential Sites |
| --- | --- | --- |
|  | Kg. Pontianak | 0 |
|  | Kg. Buau | 1 |
|  | Kg. Sulit | 1 |
|  | Kg. Pa’ Saleh | 1 |
|  | Tk. Lanting | 0 |
|  | Tg. Sauk | 1 |
|  | Tg. Lanchang | 1 |

| No | Location | Stream name | Available  head , m | Catchment area,km^2^ | River gradient |
| --- | --- | --- | --- | --- | --- |
|  | Kg. Pontianak | - | - | - | - |
|  | Kg. Buau | Unnamed (Tk. Buau) | 140 | 0.0788 | 0.229 |
|  | Kg. Sulit | Unnamed ( Tk. Sulit) | 110 | 0.03 | 0.26 |
|  | Kg. Pa’ Saleh | Unnamed (Tk. Pa’ Saleh) | 70 | 0.16 | 0.169 |
|  | Tk. Lanting | - | - | - | - |
|  | Tg. Sauk | Unnamed (Tk. Sauk) | 60 | 0.3 | 0.16 |
|  | Tg. Lanchang | Unnamed (Tk. Lanchang) | 40 | 0.15 | 0.067 |

1. **Pulau Besar/ Pulau Babi Besar**

Mountains and Hills.

- P. Babi Besar (251m) – Trigo

1. Calet P. Besar ( 2° 27’ 26” N, 103° 58’ 36”E)

- Area = ( 0.6 × 0.4)

= 0.24cm^2^

= 0.06km^2^

- Vegetation:

1. Coconut
2. Secondary jungle, Belukar, Riung and Jerami
3. Kg. Atap Zing ( 2° 26’ 19” N, 103° 58’ 42”E)

- Area = ( 0.6 × 0.6)

= 0.36cm^2^

= 0.09km^2^

- Vegetation:

1. Coconut
2. S. Tuan

- Highest elevation: 120m
- Possible intake : 60m ( 2° 26’ 11” N, 103° 59’ 05”E)
- Catchment area = (0.6 × 0.4)

= 0.24cm^2^

= 0.06km^2^

- Single line river
- Tailrace= 20m
- Available head = 60-20

= 40m

- River gradient = $\frac{60}{1.5cm \times\frac{1km}{2cm}\times\frac{1000 m}{1 km}}$ =$\frac{60}{750}$ = 0.08

1. Kg. Busong ( 2° 25’ 58” N, 103° 58’ 52”E)

- Area = ( 0.5× 1) + ( 0.5 × 0.8)

= 0.5 + 0.4

= 0.9cm^2^

= 0.225km^2^

- Vegetation:

1. Coconut
2. Kg. Kampa ( 2° 25’ 45” N, 103° 59’ 00”E)

- Area = ( 0.8 × 0.9)

= 0.72cm^2^

= 0.18km^2^

- Vegetation:

1. Coconut
2. Unnamed river ( Tk. Kampa)

- Highest elevation: 140m
- Possible intake : 60m ( 2° 25’ 50” N, 103° 59’ 15”E)
- Catchment area = (0.5 × 0.5)

= 0.25cm^2^

= 0.0625km^2^

- Single line river
- Tailrace= 20m
- Available head = 60-20

= 40m

- River gradient = $\frac{60}{0.7cm \times\frac{1km}{2cm}\times\frac{1000 m}{1 km}}$ =$\frac{60}{350}$ = 0.171

Analysis for Besar island

There are 7 location has been studied for hydropower potential assessment in Besar island

| No | Location | No. of Potential Sites |
| --- | --- | --- |
|  | Calet P. Besar | 0 |
|  | Kg. Atap Zing | 1 |
|  | Kg. Busong | 0 |
|  | Kg. Kampa | 1 |

| No | Location | Stream name | Available  head , m | Catchment area,km^2^ | River gradient |
| --- | --- | --- | --- | --- | --- |
|  | Calet P. Besar | - | - | - | - |
|  | Kg. Atap Zing | S. Tuan | 40 | 0.06 | 0.08 |
|  | Kg. Busong | - | - | - | - |
|  | Kg. Kampa | Unnamed (Tk. Kampa) | 40 | 0.0625 | 0.171 |
